# Extracellular Vesicle Dissemination of Epidermal Growth Factor Receptor and Ligands and Its Role in Cancer Progression

**DOI:** 10.3390/cancers12113200

**Published:** 2020-10-30

**Authors:** Thomas Frawley, Olga Piskareva

**Affiliations:** 1 Cancer Bio-Engineering Group, Tissue Engineering Research Group, Department of Anatomy and Regenerative Medicine, RCSI University of Medicine and Health Sciences, D02 YN77 Dublin, Ireland; 2 School of Pharmacy and Biomolecular Sciences, Royal College of Surgeons in Ireland, D02 YN77 Dublin, Ireland; 3 National Children’s Research Centre, Our Lady’s Children’s Hospital, Crumlin, D12 8MGH Dublin, Ireland; 4 Trinity Centre for Bioengineering, Trinity College Dublin, D02 8QV2 Dublin, Ireland; 5 Advanced Materials and Bioengineering Research Centre (AMBER), RCSI University of Medicine and Health Sciences and Trinity College Dublin, D02 8PVW Dublin, Ireland

**Keywords:** exosome, tumour progression, immune system, metastases, angiogenesis, osteoclastogenesis, biomarker, bio-engineering

## Abstract

**Simple Summary:**

Overexpression of the transmembrane protein, epidermal growth factor receptor (EGFR), drives tumour progression in several cancers including breast, lung, glioblastoma and head and neck cancers. In recent years, it has been shown that tumour cells can transfer EGFR to other tumour cells and non-tumour cells using extracellular vesicles (EVs). EVs are nano-sized vesicles secreted by cells and contain protein, RNA and DNA. The function of EVs is to send messages between cells which occurs in both healthy and diseased states. In this review, we will discuss how the transfer of EGFR and EGFR ligands by EVs in cancer can promote metastases, the formation of new tumour blood vessels and decrease the anti-tumour activity of immune cells. We will also discuss how EGFR contained in EVs can be used as a non-invasive diagnostic marker of cancer, and finally, how EVs can be re-engineered to promote targeting to EGFR expressing tumours.

**Abstract:**

The epidermal growth factor receptor (EGFR) pathway functions through the autocrine or paracrine activation of cellular EGFR by a number of transmembrane ligands. Amplified or mutant EGFR can lead to tumour formation due to increased cell proliferation, growth, migration and survival signals. These oncogenic effects were thought to be confined to aberrant cells hosting genetic alterations in EGFR. However, in the past decade, numerous studies identified that tumour cells could harness extracellular vesicles (EVs) to disseminate EGFR, mutant EGFR, phosphorylated EGFR and EGFR ligands to local and distant cells. This functions to impart a pro-tumourigenic phenotype in recipient cells. EVs play an essential role in intracellular communication, through receptor signalling or the release of their intra-vesicular content into recipient cells. This review will discuss the role of EVs delivering EGFR or EGFR ligands either to or from tumour cells and how this can promote metastases, pre-metastatic niche formation, osteoclastogenesis, angiogenesis and immune modulation in cancer. We will examine how circulating EVs positive for EGFR may be exploited as diagnostic, prognostic or therapeutic markers in cancers including breast, lung, glioblastoma, ovarian and prostate. Finally, we will explore recent breakthroughs in bio-engineering EVs with EGFR targeting abilities for targeted drug delivery.

## 1. Introduction

Extracellular vesicles (EVs) represent a population of cellular delivery vehicles capable of delivering complex messages through the cell-to-cell transfer of proteins, lipids and nucleic acids. This EV population is comprised of different sub-types characterised by the mode of biogenesis, size and cargo and includes apoptotic bodies, microvesicles and exosomes. The biogenesis, biology and function of EVs have been reviewed extensively elsewhere [1,2,3]. However, in brief, EVs are phospholipid bi-layer enclosed vesicles, ranging in diameter from 50 to 1000 nm that are produced in the endosomal pathway or through budding from the plasma membrane. Through mechanisms still not fully understood, whether selective or nonselective, EVs are loaded with cargo in the cell of origin, are released through budding of the plasma membrane or apposition of the multivesicular endosome to the plasma membrane. These secreted vesicles can then mediate cell–cell communication with neighbouring or distant cells through receptor signalling or release of intra-vesicular cargo into the recipient cell. This can be achieved through fusion between the EV and cellular membrane or entry of the EV into the endosomal pathway via phagocytosis, macropinocytosis or clathrin-mediated endocytosis [4].

Depending on the phenotype of the originating cell, this transfer of cargo may induce homeostatic or pathological states by activation of different pathways in the recipient cell. For example, in cancer, EVs originating from tumour cells have been shown to promote growth, metastases and drug resistance in recipient tumour cells, enhance angiogenesis in recipient endothelial cells and alter the tumour immune response in recipient immune cells [5].

**Figure 1:**
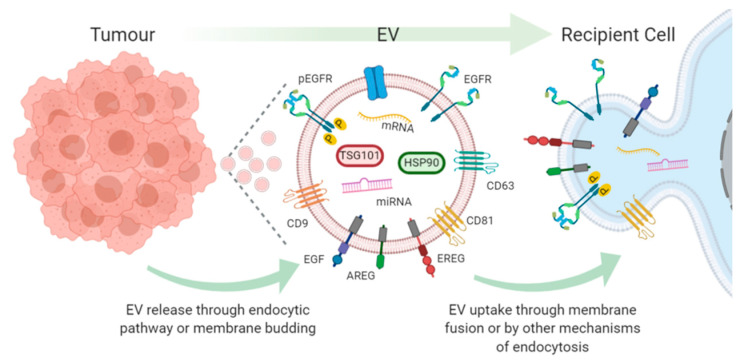
Tumour release of EVs containing EGFR monomers, phosphorylated EGFR (pEGFR), EGFR ligands; epidermal growth factor (EGF), amphiregulin (AREG), epiregulin (EREG) and subsequent uptake and transfer of cargo in recipient cell.

The canonical EGFR pathway is one of the most studied cellular signalling pathways in the context of tumour progression and targeted drug development. The EGFR pathway relies on activation of the receptor by a ligand, subsequent dimerization and signal transduction. In the past decade, a new paradigm has emerged, EV translocation of functionally active EGFR and EGFR ligands between tumour cells and local and distant cells, imparting a quasi EGFR amplified phenotype upon the recipient cell [6] (Figure 1). The effects of this new paradigm in EV EGFR signalling in cancer are now evolving, and it is beginning to provide new insights into tumour microenvironment (TME) communication and cancer progression.

The proto-oncogene EGFR, also known as ErbB1 or Her1, is a member of the ErbB family of receptor tyrosine kinases (RTK), which also includes ErbB2/Her2/Neu, ErbB3/Her3 and ErbB4/Her4 [7]. EGFR is a transmembrane protein capable of forming homodimers and heterodimers with other members of the ErbB family. Several ligands including epidermal growth factor (EGF), transforming growth factor-α, amphiregulin (AREG), epiregulin (EREG), betacellulin, heparin-binding EGF-like growth factor, and epigen regulate the EGFR function. Its activation results in signal transduction through numerous pathways leading to the stimulation of cell proliferation, differentiation, growth, migration and apoptosis inhibition [7]. Both EGFR mutations and amplifications are a driving force behind many cancers including breast, non-small cell lung carcinoma (NSCLC), glioblastoma, head and neck squamous cell carcinoma (HNSCC), ovarian and melanoma [8]. EGFR serves as a successful diagnostic and prognostic tool. It is a target for tyrosine kinase inhibitors (TKI) and monoclonal antibody therapies in cancer [8]. This review will detail the role of EVs as mediators in the EGF receptor-ligand signalling pathway and its implications in cancer progression. We will examine the prospects of utilizing EVs positive for EGFR as liquid biopsies in cancer, and finally, we explore recent developments in bio-engineering EVs with EGFR targeting abilities.

## 2. EV Transfer of EGFR and EGFR Ligands Promotes Metastases

**Figure 2:**
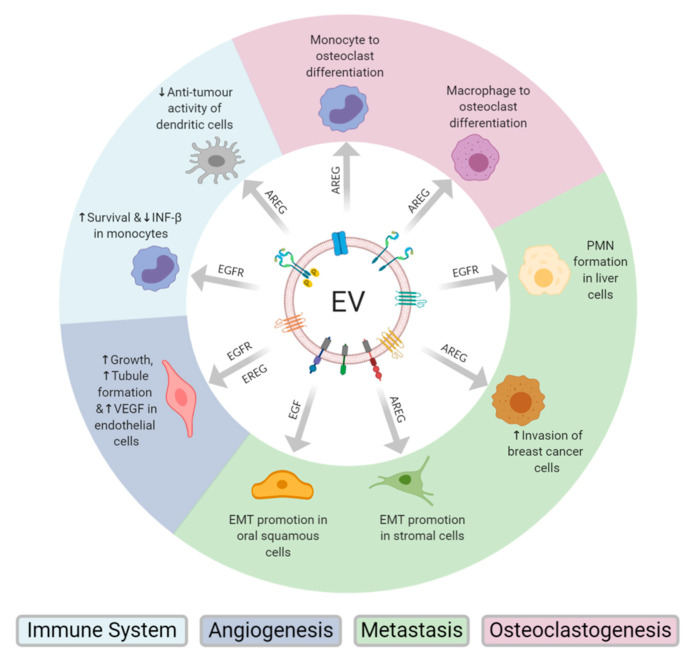
Schematic representation of the transfer of epidermal growth factor receptor, epidermal growth factor, amphiregulin or epiregulin from EVs to recipient cells of the immune system, tumour microenvironment or cells at metastatic sites. This EV-meditated transfer of EGFR or EGFR ligands has been shown to modulate the immune system, promote angiogenesis, metastasis and osteoclastogenesis in cancer.

EVs can promote metastases in many cancers by imparting migratory abilities and promoting epithelial to mesenchymal transition (EMT) in recipient cells. EVs can also play a role in pre-metastatic niche formation [9]. A growing body of evidence suggests that metastases may be driven through EV-mediated activation of the EGF receptor-ligand pathway, accomplished through tumour derived EV dissemination of EGFR, AREG or EGF to local and distant cells (Figure 2). This EV transfer of EGFR and EGFR ligands can occur in three different directions, from cancer cell to non-cancerous cell, cancer cell to cancer cell and non-cancerous cell to cancer cell.

Several independent research groups have reported cancer to non-cancerous cell transfer of EGFR. In a series of in vitro and in vivo experiments, Zhang et al. identified that tumour-derived EV EGFR activated liver hepatocyte growth factor (HGF) expression and release, through inhibition of mir-26 a/b expression, which promoted liver gastric cancer metastases in mice with increased size and weight compared to controls [6]. In vitro, EVs from SGC-7901 gastric cancer cells delivered GFP tagged EGFR to non-cancerous primary mouse liver cells resulting in a 3-fold increase in both EGFR expression and HGF secretion. This effect was reversed when EGFR was silenced in gastric cancer cell EVs. Enhanced HGF secretion strongly promoted proliferation, migration and invasion of the gastric cancer cells. These findings were replicated in vivo where mice livers were treated with HGF overexpressing lentivirus or HGF short hairpin RNA before orthotropic implant of gastric cells. The authors backed this research up with clinical data of gastric cancer patients, demonstrating upregulation of EGFR in the primary tumour, in serum EVs and liver tissue compared to controls [6].

In chronic myelogenous leukaemia (CML), the transfer of AREG between CML cells and non-cancerous stromal cells via EVs modulated the bone marrow microenvironment through EMT promotion and increased cellular adhesion of CML cells to a stromal monolayer [10]. In this study, EVs isolated from CML patients and LAMA84 CML cells were found to be enriched with AREG. LAMA84 EVs induced the activation of EGFR in HS5 stromal cells, which was significantly reduced with gefitinib co-treatment. The EMT marker SNAIL increased over 3-fold at the mRNA level, and interleukin 8 (IL-8) and matrix metalloproteinase 9 (MMP9) increased approximately 2-fold and 3-fold, respectively, at the protein level in HS5 cells after CML EV treatment. This was subsequently reduced with gefitinib and neutralizing anti-AREG antibody treatment. Similar effects were observed in primary bone marrow stromal cells treated with CML EVs [10].

Cancer to non-cancerous cell transfer of EGFR was described in oral squamous cancer cells (OSCC) in a study by Fujiwara et al. [11]. This study found that HSC-3 squamous carcinoma cell EVs containing EGFR could increase vimentin protein levels and induce a spindle shape morphology while decreasing E-cadherin levels in recipient RT7 cancer cells [11]. These EMT modulating effects were further increased with the pre-treatment of OSCC cells with EGF. Cetuximab was reported to restore vimentin levels and partially inhibit morphological changes [11]. In a subsequent publication, Fujiwara et al. demonstrated OSCC cell secretion of cetuximab via EVs, in a dose-dependent manner [12].

Higginbotham et al. captured the EV transfer of non-cancerous cell cargo to cancer cells. EVs from AREG overexpressing Madin-Darby canine kidney cells (MDCK) were found to rapidly enter human MDA231-LM2-4175 breast cancer cells, mediated in part by EGFR binding. This increased their invasiveness over 4-fold, in a Boyden cell chamber assay, compared to controls and recombinant AREG treatment [13]. The invasive potential of the breast cancer cells was reduced by 50% when EVs were treated with an anti-AREG neutralizing antibody. Similar results were obtained with human MDA-BoM-1833 triple-negative breast cancer cells [13].

## 3. Tumour Derived EV AREG Drives Osteoclastogenesis

Osteolytic bone metastasis occurs in the later stages of many cancers, whereby the bone marrow promotes metastatic growth through the interaction of metastatic cells with osteoclasts and osteoblasts which promotes bone degradation. In turn, the release of extracellular matrix-bound growth factors in the bone further supports the growth of the metastatic cells [14]. AREG containing tumour-derived EVs have been reported to induce osteoclastogenesis in both lung cancer and multiple myeloma [15,16] (Figure 2).

Taverna et al. found that an enrichment of AREG in CRL-2868 and A459 NSCLC cell-derived EVs promotes macrophage and monocyte osteoclast differentiation [15]. CRL-2868 EVs were internalized by micropinocytosis in RAW 264.7 murine macrophages inducing differentiation to mature osteoclasts, phosphorylation of EGFR, increased receptor activator of nuclear factor kappa-Β ligand (RANKL) and MMP9 protein expression by 2.5-fold and 4-fold, respectively. Increased TRAP expression was also confirmed by microscopy. A similar response was seen in primary human monocytes. EVs with decreased AREG, AREG neutralizing antibody, and erlotinib treatment reverted the osteoclast differentiating effect of native EVs. These findings were further supported by the ex vivo osteoclast differentiation of human primary monocytes with patient-derived NSCLC EVs through AREG [15].

The osteoclast differentiating effects of AREG-enriched EVs were further supported by a study by Raimondo et al. in multiple myeloma [16]. In this study, AREG enriched MM1.S multiple myeloma cell and multiple myeloma patient-derived bone marrow EVs promoted both RAW 246.7 and primary human CD14^+^ cell osteoclast differentiation, through EGFR activation, a 1.5-fold increase in MM9 protein expression and an increase in TRAP staining. Furthermore, MM1.S EVs induced the activation of EGFR, decreased the gene expression of osteoblast markers *ALP, OCN* and *COL1 A1* by at least 2-fold and increased production and secretion of pro-osteoclastogenic cytokine IL-8 over 3-fold in mesenchymal stromal cells. This osteoclast differentiating effect subsequently promoted the adhesion of MM1.S cells to a mesenchymal stromal cell monolayer. These osteoclast differentiating effects were reversed by AREG neutralizing antibody treatment [16].

## 4. Endothelial Cell Activation through EV Transfer of EGFR and EREG

EVs may contain many angiogenic factors including angiogenin, vascular endothelial growth factor (VEGF), annexin A2, transforming growth factor-β (FGF-β) and basic fibroblast growth factor. Tumour-derived EVs have been shown to promote angiogenesis in cancers such as myeloma, ovarian, prostate, breast and glioblastoma [17]. Additionally, some evidence suggests that tumour-derived EVs containing EGFR and EREG may also promote tumour angiogenesis (Figure 2).

Al-Nedawi et al. demonstrated the translocation of EGFR from A431 cells to human umbilical vein endothelial cells and human microvascular endothelial cells via EVs in a dose-dependent manner [18]. EV transfer of EGFR activated MAPK and Akt in endothelial cells, which was inhibited by pre-treatment with annexin V, a pan-ERB kinase inhibitor and an anti-EGFR neutralizing antibody. EV treatment increased VEGF secretion approximately 2-fold and increased the growth and viability of the endothelial cells, which was reversed with the pre-treatment with annexin V and pan-ERB kinase inhibitor. Daily injections of Diannexin, to inhibit EV communication, reduced tumour growth by 57% and microvascular densities by 37% in A431 xenografts in SCID mice [18].

A study by Yang et al. described endothelial tubule formation in vitro mediated via EREG-enriched EVs secreted by SACC-83 and SACC-LM salivary adenoid cystic carcinoma cells [19]. Overexpression of EREG in EVs enhanced tubule formation in endothelial cells, which was reversed with siRNA knockdown of EREG in EVs. Human pulmonary microvascular endothelial cells treated with SACC-83 EVs increased mRNA levels of *VEGF-A, FGF-β* and *IL-8* approximately 1.5-fold, which was further increased with overexpression of EREG in EVs. In vitro data was backed up by in vivo studies demonstrating a 3-fold increase in the size of lung metastases, in NOD SCID mice after intravenous injection of EREG-enriched EVs. CD146^+^ sorted endothelial cells from mice treated with EREG expressing EVs had at least 3-fold higher levels of *VEGF-A, FGF-β* and *IL-8* mRNA compared to mice treated with native EVs [19].

## 5. Tumour-Derived EV EGFR Modulation of the Immune Response

Evidence is emerging that tumour-derived EVs can have an immune-modulating effect, mediated through the EGFR, in both lung and breast cancer. In lung cancer, tumour-derived EVs carrying EGFR decreased the IFN-β response in monocytes and macrophages and stimulated a dendritic cell-mediated immunosuppressive tumour microenvironment [20,21,22]. In breast cancer, tumour-derived EVs promoted monocyte survival, in a pro-inflammatory environment, through EGFR [23] (Figure 2).

Gao et al. found that EVs containing EGFR are increased in lung cancer patients and are inversely correlated with circulating INF-β, a type 1 interferon with antiviral activity [20]. Co-culture of A549 EVs with human THP-1 monocytes and murine RAW264.7 macrophages dramatically increased EGFR levels in these immune cells, suggesting translocation of tumour-derived EV EGFR. Lewis lung cancer (LLC) xenograft model infected with vesicular stomatitis virus or HSV-1, had an approximately 2-fold reduction in serum IFN-β compared to non-inoculated mice. Doxycycline repression of EGFR expression in LLC tumours restored normal levels of circulating INF-β and suppressed viral titres. This study also demonstrated that the suppression of INF-β production was the result of tumour-derived EV EGFR activation of MEKK2 in the recipient macrophages [20].

A study by Huang et al. in 2013 documented the induction of dendritic cell (DCs) differentiation to tolerogenic DCs mediated through lung cancer tumour-derived EVs carrying EGFR. EVs isolated from tumour tissue of lung cancer patients and lung tissue from patients with chronic inflammation were found to be 80% and 2% positive for EGFR staining, respectively [21]. Treating patient-derived DCs with EVs from lung cancer patients induced the accumulation of indoleamine 2,3-dioxygenase (IDO), a marker of tolerogenic DCs, in 80% of cells, compared to 65% of DCs treated with EVs from lung cancer patients with chronic inflammation. This induction of IDO was mediated through PI3K and abrogated with an EGFR neutralising antibody. IDO^+^ DCs converted CD4^+^ T cells to regulatory T cells, which suppressed the anti-tumour functions of CD8^+^ T cells [21]. This study, published in the early stages of EV research, does not provide any information on the size range or protein content of isolated EVs and most importantly, no analysis was performed on possible non-EV contaminants introduced during tissue homogenisation and subsequent EV isolation.

A 2020 study by Yu et al. also reported that EVs from EGFR mutant lung cancer cells could control DCs to repress anti-tumour immunity development [22]. Firstly, they identified an increase of CD8^+^ T cells in EGFR wild-type patient tumours compared to tumours harbouring the EGFR-19 del mutation. Mice with EGFR-19 del LLC cell tumours had lower numbers of CD8^+^ and CD4^+^ cells. These mice also had different DC subtypes in their draining lymph nodes, that partially lost their ability to stimulate proliferation of naive T cells, compared to mice with EGFR wild-type tumours. Inhibiting EV release, reversed the effect of EGFR-19 del LLC cells immunosuppressive effect on DCs. In vitro, EVs from EGFR-19 del LLC cells transferred EGFR-19 del mRNA and protein to DCs. Increased tumour size, less necrosis and reduced numbers of CD8^+^ Ki67 ^+^ and CD4^+^ IFN-γ^+^ cells were observed in mice injected with EGFR-19 del LLC EVs compared to wild-type EVs. Taken together, this evidence suggests the EGFR-19 del lung cancer cells can transmit their EGFR phenotype to DCs, resulting in an immunosuppressive tumour microenvironment [22].

Breast cancer cell EVs have been shown to promote monocyte survival through EGFR transfer and subsequent activation of the MAPK pathway [23]. In this study, MCF-7 breast cancer cells enriched in phosphorylated EGFR and HER2, cultured in a pro- (LPS + INF-γ) or conflicting- (LPS + INF-γ + IL-4) inflammatory environment, released EVs that increased the survival and viability of primary human monocytes. Knockdown of EGFR or HER2 in MCF-7 cell-derived EVs abrogated this EV-mediated survival in primary monocytes. This was reported to be propagated through EGFR activation of ERK, which lead to a decrease in caspase-8 activation in the primary monocytes [23].

## 6. EV Transfer of EGFRvIII Leads to Transformation of Cellular Phenotype

EGFRvIII is a constitutively activated mutant variant of EGFR that is found in glioblastoma multiforme (GBM), breast cancer, NSCLC and HNSCC [24]. The EV transfer of EGFRvIII was first demonstrated by Rak et al. in U373 glioma cells [25]. DNA vector-mediated EGFRvIII expression in U373 cells (U373 vIII) resulted in the incorporation of EGFRvIII into their EVs. The transfer of GFP tagged EGFRvIII to U373 cells via EVs was confirmed by fluorescence-activated cell sorting analysis.

This transfer resulted in a nearly 2-fold increase of Erk 1/2 phosphorylation in U373 cells compared to treatment with native U373 EVs. Pre-treatment of U373 vIII EVs with a pan-ERB kinase inhibitor and annexin V reversed this phosphorylation. Similar results were seen with Akt, PDK1 and Raf. Additionally a 2 to 3-fold increase in VEGF production was identified in U373 cells exposed to EGFRvIII containing EVs, which was subsequently reduced with a pan-ERB inhibitor. Evidence of cellular phenotypic changes were apparent with an increase in spindle-like morphology and a 2-fold increase in anchorage-independent colony formation of U373 cells treated with U373 vIII EVs [25].

In a subsequent study, Rak et al. showed that EGFRvIII expression in U373 cells alters the proteome of U373-derived EVs [26]. Mass spectrometry of U373 and U373 vIII-derived EVs identified 1059 proteins of which 4.2% were unique to U373 EVs and 9.2% to U373 vIII EVs. Gene ontology analysis of significantly upregulated proteins in U373 vIII EVs revealed enrichment of proteins involved in cellular adhesion, actin cytoskeleton regulation and extracellular-matrix-related proteins [26].

## 7. Activation of EGFR Through Exomeres Carrying AREG

Exomeres are a recently discovered subpopulation of EVs, they are non-membranous vesicles approximately 35 nm in diameter and have a distinct proteomic cargo compared to exosomes [27]. Zhang et al. discovered that exomeres contain AREG that can activate the EGFR pathway in recipient cells [28]. Exomeres isolated from MDCK cells by differential centrifugation were between 39–71 nm in size when measured by nanoparticle tracking analysis and <50 nm by transmission electron microscopy. Exomeres from MDCK cells and AREG overexpressing MDCK cells both contained AREG and transferred AREG to DiFi rectal carcinoma cells, resulting in phosphorylation of EGFR in the recipient cells. AREG overexpressing MDCK exomeres were also found to increase the size and number of colonic tumour organoids [28].

## 8. EV EGFR as a Potential Biomarker in Cancer

Circulating EVs have shown great promise as a non-invasive surrogate source of genetic and phenotypic tumour information. Their use as diagnostic, prognostic and therapeutic marker has been explored in many different cancers [29,30]. EGFR DNA, RNA and protein contained in tumour-derived EVs are now being investigated as potential biomarkers for cancers including breast, glioblastoma, lung, ovarian and prostate (Table 1).

The use of a microfluidic electrochemical immunosensor successfully facilitated the detection of EGFR protein in EVs [31]. EV proteins isolated from the plasma of 30 pre-operative breast cancer patients and 20 healthy patients were introduced into the immunosensor followed by HRP-conjugated anti-EGFR antibody. 4-tert-Butylcatechol mediated an enzymatic reaction, and the resulting current was measured to give an indirect measurement of EGFR levels in the sample. EV EGFR levels were on average over eight times higher in breast cancer patients compared to healthy patients. This assay was 80% sensitive and 90% specific in the diagnosis of breast cancer. EV EGFR levels significantly correlated with tumour EGFR levels, tumour size and Ki67 tumour expression, suggesting that this microfluidic electrochemical immunosensor for EV EGFR may be useful in early diagnosis of breast cancer patients [31].

Another approach to identify and quantify EGFR positive EVs utilises aldehyde beads and an anti-EGFR antibody coupled with flow cytometry. Using this method, significantly higher levels of EV EGFR were identified in 23 glioma patients compared to 12 healthy patients [34]. The evaluation of EV EGFR levels in glioma patients was 86.96% sensitive and 83.7% specific in identifying patients with glioma. EV EGFR levels positively correlated with tumour grade and with Ki67 tumour expression. Surgical resection of the glioma dramatically decreased EV EGFR levels [34].

EV cargo presents an alternative source for EGFR genotyping. This is typically performed on cells or cell blocks from pleural effusions in pulmonary adenocarcinoma with pleural effusion. Lee et al. carried out a comparative study to determine if pleural effusion EVs would be a better source for EGFR genotyping of a panel of 29 EGFR mutations [42]. Out of 32 EGFR TKI-naive patients, with known EGFR tissue genotype, EV EGFR genotyping in pleural effusions correctly identified 19 EGFR mutant cases. EV EGFR genotyping identified 3 additional EGFR mutants in the remaining 13 cases previously identified as EGFR wild-type by tissue genotyping. T790 M genotyping of pleural effusion EVs in 18 patients, with acquired TKI resistance, was 100% in accordance with tissue T790 M status and outperformed cell block genotyping which only identified three T790 M positive cases [42].

The listed studies were performed on a limited sample size ranging from 9 to 161 patients (mean = 58). Before this research progresses to more extensive analytical and clinical validation studies, a number of issues need to be addressed. Currently, there is little consensus on the appropriate EV population to isolate, which EV isolation method to use and how pure this isolation needs to be. In the 14 studies outlined in Table 1, 6 different methods of EV isolation were performed, isolating differing EV populations with varying degrees of purity. Adequate EV characterisation also needs to be performed, following the Minimal information for studies of extracellular vesicles 2018 (MISEV2018) guidelines [45], to identify the population of EV and to identify potential non-EV contaminants introduced during isolation. EV characterisation was not fully performed in 4 of the 16 studies outlined in Table 1.

## 9. Bio-Engineering EVs to Enhance EGFR Targeting

EV based drug delivery systems have numerous benefits due to their physical properties, low immunogenicity, biocompatibility and stability in circulation [46]. An ability to target tumours specifically would add great value to this system. The EGFR is a driver behind some solid tumours including lung, breast and glioblastoma and serves as a highly attractive target for bio-engineering EVs with EGFR targeting abilities [47]. In this section, we will discuss the most exciting directions in this field.

**Figure 3:**
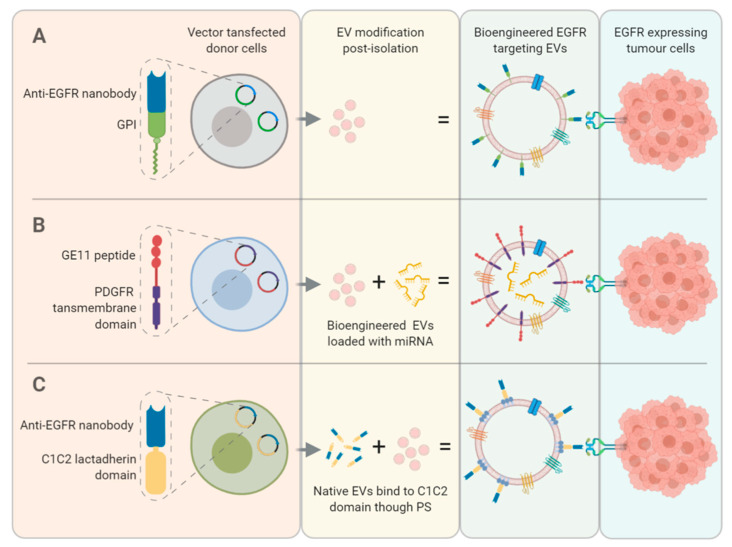
Bio-engineering strategies to enhance EV targeting to EGFR expressed on tumour cells include: (**A**) transfection of a donor cell with a plasmid containing anti-EGFR nanobody and glycosylphosphatidylinositol (GPI) elements and (**B**) a plasmid containing GE11 peptide and platelet derived growth factor receptor (PDGF) elements which would subsequently be packaged into the donor cell derived EVs. (**C**) Native EVs bind to the C1C2 domain of a recombinant protein containing an anti-EGFR nanobody through phospholipid phosphatidylserine (PS).

Kooijmans et al. engineered EVs with single-domain antibodies, or nanobodies, to target EGFR expressing tumour cells [48] (Figure 3). These anti-EGFR nanobodies anchored to glycosylphosphatidylinositol on the EV membrane were expressed by a vector transfected into Neuro2 A mouse neuroblastoma cells. The bio-engineered EVs facilitated EGFR cell binding in an EGFR-dependent manner that was replicated under flow conditions with a live-cell perfusion experiment. Using this bio-engineering method, EVs loaded with therapeutic agents would have enhanced tumour targeting abilities.

Furthermore, Kooijmans et al. improved EV targeting through EGFR by exploiting the properties of phospholipid phosphatidylserine (PS) expressed on the external leaf of the EV membrane to plug and play EVs with EGFR targeting abilities post isolation [49] (Figure 3). An anti-EGFR nanobody with a C1C2 lactadherin domain was expressed and isolated from HEK293, human embryonic kidney cells, and then incubated with red blood cell and Neuro2 A cell-derived EVs. These nanobodies exclusively bound to PS on the EVs membrane and did not affect EV size or morphology. The uptake of the bio-engineered EVs was measured in EGFR negative Neuro2 A cells and EGFR overexpressing A431 cells. Compared to native EVs, no uptake was seen in Neuro2 A cells, whereas significant uptake was seen in A431 cells [49].

Ohno et al. engineered HEK293 EVs to express the transmembrane domain of platelet-derived growth factor receptor with the EGFR targeting peptide ligand GE11 [50] (Figure 3). The degree of GE11 EV binding reflected EGFR expression in a panel of cells and did not alter their proliferation. These bio-engineered EVs were loaded with tumour suppressor miRNA, let-7a, by lipofection and injected into mice bearing luciferase-expressing HCC70 cell tumours. Three times more GE11 EVs containing let-7a were detected in the cell tumour, suggesting EV targeting to tumour expressed EGFR, which significantly suppressed tumour growth compared to control EVs [50].

In EV-derived therapeutics, bio-engineering EVs with enhanced tumour targeting abilities to deliver EV bound therapeutic agents, has been the main focus. Xie et al. have inverted this paradigm by proposing and developing a method to target, bind to and remove tumour-derived EVs expressing EGFR from circulation through hepatobiliary and intestinal excretion [51]. This could, in theory, reduce or minimize the oncogenic role of EVs in cancer. In this study, mesoporous silica nanoparticles (MSN) were functionalised with EGFR targeting aptamers (MSN-AP) to recognise and bind to A549 lung cancer cell-derived EVs in both static and rocking flow conditions. A549 EVs with high EGFR levels and control EVs with low EGFR levels were injected into the portal vein of mice followed by local injection of MSN-AP. These nanoparticles significantly enhanced A549 EV levels in the duodenum compared to controls, suggesting that MSN-AP could bind to EGFR EVs and tow them across the intestinal wall into the duodenum for excretion. MSN-AP with EV EGFR targeting ability inhibited lung-specific metastases and eliminated oncogenic EVs from mice bearing A549 cell tumours [51].

## 10. Conclusions

Over the last decade, it has become clear that tumour cells can appropriate EV communication to bombard cells of the TME, distant cells and other tumour cells with complex heterogenic messages to promote cancer progression. Decoding these complex messages has identified EGFR and EGFR ligands as essential mediators in this process.

It appears that tumour cells use EVs as a mechanism to transfer EGFR across a gradient, from cells with high EGFR to cells with lower EGFR expression. This was demonstrated in a number of studies, whereby we see the transfer of EGFR from lung cancer cells to endothelial cells, dendritic cells, macrophages and monocytes, activating pro-tumourigenic pathways in these low EGFR expressing recipient cells.

EGFR ligands normally function as autocrine or paracrine activators of EGFR; however, by packaging EVs with EGFR ligands, tumours have found a mechanism to extend the functional distance these ligands can act upon and possibly to target specific cells with larger concentrations of EGFR ligands. This was partly demonstrated by Yang et al., where EREG overexpressing EVs intravenously introduced into mice significantly increased the number of lung metastases of SACC cells [19].

An important question that remains unanswered, is do tumour cells know what cargo they are loading into EVs? If the answer is yes, then there is likely to be a molecular mechanism that actively sorts specific cargo like EGFR, AREG and EGF into EVs and a mechanism that actively prevents loading of other cargo. If we can answer this question, then developing a therapy to prevent tumours from loading EVs with pro-oncogenic cargo will become a distinct possibility.

EVs offer a proverbial treasure chest of tumour information that is non-invasive and easily accessible from many biological sources including serum, plasma, CSF, BALF and pleural effusions. EV EGFR is now being explored as an alternative diagnostic and prognostic marker to tissue EGFR in cancers including breast, lung, glioblastoma, ovarian and prostate (Table 1). This has numerous advantages; it is non-invasive; it may allow for real-time monitoring of response to treatment and EVs may contain other biomarkers to assist in prognosis, and treatment stratification.

This mechanism of cancer cell signalling represents a new opportunity in cancer research to understand better how tumours communicate with the tumour microenvironment and distant cells. It provides an opportunity to develop better diagnostic and prognostic tools, to create new treatments to circumvent the growing problem of treatment resistance and to create a new method of more effectively delivering cancer therapies to the tumour site.

## Figures and Tables

**Table 1:** Liquid biopsies and EV EGFR.

Cancer	EV Type	Biomolecule	EGFR Biomarker	EV Source	Isolation/Detection Method	Ref.
Breast	Small EVs	Protein	WT	Plasma	DC; Immunosensor	[31]
Glioblastoma	EVs *	RNA	WT, EGFRvIII	CSF	DC; PCR	[32]
Glioblastoma	Small EVs	RNA	WT, EGFRvIII	Plasma	Total Exosome Isolation Kit; PCR	[33]
Glioblastoma	Small EVs	Protein	WT	Serum	DC; Flow cytometry	[34]
Glioblastoma	Small EVs	Protein	WT, EGFRvIII	Serum	DC; Electrochemical biosensor	[35]
Lung	Small EVs	Protein	WT	Plasma	Dielectrophoretic chip; Immunofluorescence	[36]
Lung	EVs	RNA	T790 M	Plasma	NanoVilli Chip; PCR	[37]
Lung	EVs *	DNA/RNA	Ex19 del, T790 M	Plasma	ExoQuick; PCR	[38]
Lung	EVs *	DNA	WT, EGFR mutation panel	Pleural Effusions	ExoQuick; PCR	[39]
Lung	EVs *	DNA/RNA	WT, EGFR mutation panel	Plasma	Exolution; PCR	[40]
Lung	Medium EVs	DNA	WT, EGFR mutation panel	BALF	DC; PCR	[41]
Lung	Medium EVs	DNA	WT, EGFR mutation panel	Pleural Effusions	DC; PCR	[42]
Ovarian	Small EVs	Protein	WT	Plasma	DC; Microfluidic device	[43]
Prostate	Small EVs	Protein	WT	Plasma	DC; ELISA	[44]

Abbreviations: Small EVs = EVs <200 nm, Medium EVs = EVs <500 nm, EVs = EVs <1000 nm, WT = wild-type, CSF = Cerebrospinal fluid, BALF = Bronchoalveolar lavage fluid, DC = Differential centrifugation, T790M = threonine to methionine missense substitution at position 790 in exon 20 of EGFR, Ex19 del = in-frame deletion in exon 19 of EGFR. * EV characterisation not performed to MISEV2018 guidelines; no information on EV size range.
